# Membrane Raft Redox Signaling Mediates Trimethylamine N-Oxide-Induced NLRP3 Inflammasome Activation and Endothelial Dysfunction

**DOI:** 10.3390/antiox15091138

**Published:** 2026-09-09

**Authors:** Md Areeful Haque, Mohammad Atiqur Rahman, Inavolu Sriram Sandeep, Sindhura Pasham, Sayantap Datta, Krishna M. Boini, Saisudha Koka

**Affiliations:** Department of Pharmacological and Pharmaceutical Sciences, College of Pharmacy, University of Houston, Houston, TX 77204, USA

**Keywords:** TMAO, NLRP3 inflammasome, membrane rafts, NADPH oxidase, endothelial dysfunction

## Abstract

Trimethylamine-N-oxide (TMAO), a gut microbiota-derived metabolite, is a known risk factor for cardiovascular disease. We previously showed that TMAO induces NLRP3 inflammasome activation and contributes to endothelial dysfunction. However, the upstream mechanisms linking TMAO to inflammasome activation and barrier injury remain unclear. In the present study, we examined whether membrane raft (MR) redox signaling contributes to TMAO-induced NLRP3 inflammasome activation and endothelial dysfunction. MR clustering, colocalization of MRs with NADPH oxidase subunits, inflammasome formation, and junction protein expression were assessed by Western blot analysis. RT-qPCR was performed to further assess the mRNA expression of junctional genes. Superoxide production was measured by electron spin resonance (ESR), caspase-1 activity was determined using a biochemical assay kit, IL-1β production was measured by ELISA, and endothelial permeability was evaluated using an FITC-dextran assay. TMAO increased MR clustering in endothelial cells in a dose-dependent manner. TMAO also enhanced the colocalization of MRs with p47phox and gp91phox, indicating the formation of an MR-associated redox signaling axis. In addition, TMAO increased superoxide production; promoted NLRP3 inflammasome formation; elevated caspase-1 activity and IL-1β production; reduced the expression of ZO-2, ZO-1, VE cadherin and occludin; and increased endothelial permeability. Pretreatment with the MR disruptor MCD, the NADPH oxidase inhibitor DPI, or the caspase-1 inhibitor WEHD significantly attenuated these TMAO-induced effects. These findings demonstrate that TMAO-mediated endothelial injury is dependent on the MR-associated redox signaling axis. TMAO promotes MR-associated redox signaling, leading to NADPH oxidase activation, superoxide generation, NLRP3 inflammasome activation, disruption of endothelial junction proteins, and barrier dysfunction. Targeting MR-associated signaling pathways may offer a novel therapeutic strategy to mitigate TMAO-induced vascular inflammation and endothelial dysfunction.

## 1. Introduction

The endothelium plays a central role in maintaining vascular homeostasis by regulating barrier integrity, inflammation, and oxidative stress [1,2]. Endothelial dysfunction is a hallmark of many cardiovascular and metabolic diseases and is characterized by increased vascular permeability, inflammation, and tissue injury [3,4]. Emerging evidence has linked gut microbiota-derived metabolites, such as trimethylamine-N-oxide (TMAO), to endothelial dysfunction and heightened cardiovascular risk. TMAO is a gut microbiota-derived metabolite that has attracted considerable attention for its role in the development and progression of cardiovascular diseases [5,6,7]. It is generated from dietary precursors such as choline, phosphatidylcholine, and carnitine, which are metabolized by gut microbes into trimethylamine (TMA) and subsequently oxidized in the liver by flavin-containing monooxygenase 3 (FMO3) to form TMAO [8,9]. Elevated plasma levels of TMAO have been consistently associated with an increased risk of major adverse cardiovascular events, including myocardial infarction, stroke, and mortality [10]. Mechanistically, TMAO contributes to vascular inflammation and oxidative stress, thereby promoting endothelial dysfunction, a key early event in atherosclerosis and other cardiovascular disorders [11,12]. TMAO also enhances foam cell formation by altering cholesterol and bile acid metabolism, thus accelerating atherogenesis [13]. In addition, TMAO promotes platelet hyperreactivity, increasing the risk of thrombosis and adverse cardiovascular outcomes [14]. This dual effect of TMAO on promoting vascular inflammation and coagulation underscores its critical role in the pathophysiology of cardiovascular diseases.

The membrane raft (MR) redox signaling pathway plays a critical role in cellular signaling and pathophysiological processes, particularly in endothelial dysfunction [15]. MRs are specialized microdomains within the plasma membrane enriched with cholesterol, sphingolipids, and proteins, which serve as platforms for signal transduction [16]. These domains facilitate the clustering and activation of redox enzymes, such as NADPH oxidase, thereby amplifying reactive oxygen species (ROS) production [17]. In endothelial cells, MR-associated redox signaling has been linked to oxidative stress, inflammatory responses, and disruption of barrier integrity [18,19]. MR redox signaling has been implicated in several pathological conditions, including atherosclerosis, diabetes, and vascular inflammation, suggesting that it may represent a potential therapeutic target in endothelial dysfunction and related vascular diseases [16]. Because MRs concentrate signaling molecules within discrete membrane domains, they may facilitate the assembly of redox and inflammatory signaling complexes in response to metabolic stress.

A key mediator of endothelial dysfunction is the activation of NADPH oxidase, an enzyme complex responsible for the generation of ROS [20]. NADPH oxidase-derived superoxide (O_2_·^−^) contributes to oxidative stress, which, in turn promotes inflammasome activation and the disruption of endothelial tight and adherent junctions [18,21]. Among inflammasomes, the NLRP3 inflammasome has gained significant attention due to its role in mediating inflammatory responses through the activation of caspase-1 and the release of pro-inflammatory cytokines like interleukin-1β (IL-1β) [22,23,24]. Our research group has previously established that TMAO induces NLRP3 inflammasome activation, contributing to endothelial dysfunction and potentially driving the initiation of atherogenesis [25]. However, the upstream mechanisms by which TMAO promotes NLRP3 inflammasome activation and endothelial injury remain unclear. Emerging evidence suggests that MR domains, as platforms for signal transduction, play a pivotal role in orchestrating redox signaling and inflammasome activation in response to cellular stressors [26,27]. However, the role of the MR redox signaling pathway in TMAO-induced NLRP3 inflammasome activation has not been fully defined. In addition, the contribution of downstream caspase-1 activation to TMAO-induced endothelial barrier disruption remains to be clarified. In this study, we hypothesized that TMAO activates the NLRP3 inflammasome through an MR-dependent redox signaling mechanism. Specifically, we proposed that TMAO promotes MR clustering, leading to the assembly and activation of NADPH oxidase complexes and subsequent ROS generation. This redox signaling cascade may enhance inflammasome assembly and caspase-1 activation, resulting in inflammatory cytokine production, loss of junction protein expression, and impairment of endothelial barrier function.

## 2. Methods

### 2.1. Cell Culture

The mouse endothelial cell line (EOMA) was purchased from ATCC (Manassas, VA, USA; CRL-2586), which was isolated originally from mouse hemangioendothelioma. These EOMA cells were cultured in Dulbecco’s Modified Eagle Medium (DMEM; Gibco, Carlsbad, CA, USA) supplemented with 10% fetal bovine serum (FBS; Thermo Fisher Scientific, Waltham, MA, USA) and 1% penicillin–streptomycin (Thermo Fisher Scientific, Waltham, MA, USA) [28]. Cells were maintained in a humidified incubator at 37 °C with 95% air and 5% CO_2_ and were passaged using trypsin-EDTA (Sigma, St. Louis, MO, USA) upon reaching confluence. For experimental treatments, EOMA cells were pre-treated with the membrane raft disruptor methyl-β-cyclodextrin (MCD), the NADPH oxidase inhibitor diphenyleneiodonium (DPI), or the caspase-1 inhibitor WEHD prior to stimulation with TMAO (60 μM). Following pre-treatment, cells were incubated overnight with TMAO in the continued presence or absence of the respective inhibitors. The concentrations of TMAO and inhibitors, as well as the duration of treatments, were optimized based on prior studies and preliminary experiments.

### 2.2. Immunofluorescence Staining and Confocal Microscopy

To visualize membrane rafts (MRs) and proteins associated with inflammasome formation, EOMA cells were cultured on poly-L-lysine-coated chambers and treated overnight with TMAO, DPI, MCD, or WEHD. After treatment, cells were rinsed with cold PBS and fixed with 4% paraformaldehyde for 10 min. Cells were then permeabilized with 0.1% Tween-20 in PBS for 10 min and blocked with 1% BSA in PBS for 1 h. For analysis of MR colocalization with NADPH oxidase subunits, cells were incubated overnight with anti-gp91phox (1:100, Santa Cruz Biotechnology, Dallas, TX, USA; Cat. No. sc-130543) and anti-p47phox (1:100, Santa Cruz Biotechnology, Dallas, TX, USA; Cat. No. sc-17845) primary antibodies, followed by Alexa Fluor 555-conjugated secondary antibodies (1:500, Invitrogen, Carlsbad, CA, USA) for 1 h. For MR staining, Alexa Fluor 488-labeled cholera toxin B (CTXB; 1 μg/mL, Thermo Fisher Scientific, Waltham, MA, USA; Cat. No. C34775) was applied for 2 h at 4 °C. For inflammasome analysis, cells were incubated with anti-NLRP3 (1:100, Abcam, Waltham, MA, USA; Cat. No. ab4207) and anti-ASC (1:100, Santa Cruz Biotechnology, Dallas, TX, USA; Cat. No. sc-514414) or anti-NLRP3 (1:100, Abcam, Waltham, MA, USA) and anti-caspase-1 (1:100, Santa Cruz Biotechnology, Dallas, TX, USA; Cat. No. sc-56036) primary antibodies. Secondary labeling was then performed using Alexa Fluor 488- and Alexa Fluor 555-labeled secondary antibodies (1:500, Invitrogen, Carlsbad, CA, USA; Cat. Nos. A-11055, A-21428, A-31572, A-31570) for 1 h. After washing, slides were mounted using a DAPI-containing mounting medium (Vector Laboratories, Newark, CA, USA). Images were acquired using a Nikon AXR confocal microscope system (Nikon Instruments Inc., Melville, NY, USA). Colocalization was quantified by calculating the Pearson correlation coefficient (PCC) using ImageJ software (version 1.54g) for NLRP3/ASC, NLRP3/caspase-1, and lipid raft/gp91phox or p47phox colocalization [25,29,30,31].

### 2.3. Electron Spin Resonance (ESR) Analysis of (O_2_·^−^) Production

Superoxide (O_2_·^−^) production was measured using electron spin resonance (ESR). Proteins extracted from EOMA cells were resuspended in a modified Krebs-HEPES buffer containing 100 mM deferoxamine and 5 mM diethyldithiocarbamate (Sigma-Aldrich, St. Louis, MO, USA). To evaluate NADPH-dependent O_2_·^−^ production, 1 mM NADPH was added to 20 µg of protein, and the mixture was incubated at 37 °C for 15 min in the presence or absence of 200 U/mL superoxide dismutase (SOD). After incubation, 1 mM of the superoxide-specific spin trap 1-hydroxy-3-methoxycarbonyl-2,2,5,5-tetramethylpyrrolidine (CMH; Noxygen, Elzach, Germany) was added to the reaction mixture. Samples were then loaded into glass capillaries, and O_2_·^−^ generation was analyzed for 10 min using a Miniscope MS5000 ESR spectrometer (Magnettech Ltd., Berlin, Germany) [32]. NADPH-dependent superoxide production was expressed as fold change relative to the control group.

### 2.4. Caspase-1 Activity and IL-1β Production

To evaluate the effects of TMAO and its combination treatments with the MR disruptor MCD, the NADPH oxidase inhibitor DPI, or the caspase-1 inhibitor WEHD, EOMA cells were harvested following treatment. Cell lysates were prepared and used to measure caspase-1 activity using a commercially available caspase-1 activity assay kit (Abcam, Waltham, MA, USA) [31]. Caspase-1 activity was expressed as fold change relative to untreated control group. For measurement of IL-1β production, culture supernatants were collected from treated cells, and IL-1β levels were determined using a mouse IL-1β ELISA kit (R&D Systems, Minneapolis, MN, USA) according to the manufacturer’s instructions [31].

### 2.5. Western Blot Analysis

EOMA cells were harvested after the indicated treatments, and total protein was extracted from cell lysates. Equal amounts of protein were separated by SDS-PAGE and transferred onto polyvinylidene difluoride membranes. After blocking with 5% non-fat milk or BSA in TBST for 1 h at room temperature, membranes were incubated overnight at 4 °C with primary antibodies against ZO-2 (Invitrogen, Carlsbad, CA, USA; Cat. No. 71-1400) or VE-cadherin (Abcam, Waltham, MA, USA; Cat. No. ab33168). The membranes were then washed and incubated with appropriate horseradish peroxidase-conjugated secondary antibodies for 1 h at room temperature. Immunoreactive bands were detected using enhanced chemiluminescence and visualized with an Odyssey chemiluminescence imaging system (LI-COR Inc., Lincoln, NE, USA). Following detection of the target protein, membranes were stripped using Restore™ PLUS Western Blot Stripping Buffer (Thermo Fisher Scientific, Waltham, MA, USA) for 15 min at room temperature, reblocked for 30 min, and subsequently reprobed with the indicated primary antibodies. β-Actin was used as a loading control where appropriate. Band intensities were quantified using ImageJ software (version 1.54g), and target protein expression was normalized to β-actin and expressed as fold change relative to untreated control cells.

### 2.6. RT-qPCR

Total RNA was isolated from EOMA cells after the indicated treatments using TRIzol reagent (Thermo Fisher Scientific, Waltham, MA, USA) according to the manufacturer’s instructions. RNA concentration and purity were assessed by measuring absorbance at 260 and 280 nm. Equal amounts of total RNA were reverse-transcribed into cDNA using a reverse transcription kit according to the manufacturer’s protocol (Bio-Rad, Hercules, CA, USA). Quantitative real-time PCR was performed using SYBR Green PCR Master Mix on a CFX Connect real-time PCR detection system (Bio-Rad, Hercules, CA, USA). The primer sequences were as follows: ZO-1 forward, 5′-GGATGGTGCTACAAGTGATGA-3′ and reverse, 5′-GTGTCTACTGTCCGTGCTATAC-3′; ZO-2 forward, 5′-CGACTCTGAAGTGGAGGATA-3′ and reverse, 5′-TCTCAGTGGAGGAATGGTAA-3′; occludin forward, 5′-GAGCTTACAGGCAGAACTAGAC-3′ and reverse, 5′-CAGCCATGTACTCTTCACTCTC-3′; VE-cadherin forward, 5′-CTGTAGGGAAAGAGTCCATTGT-3′ and reverse, 5′-GCAGCATTCTCACACACTTTAG-3′. β-actin was used as internal control. Amplification was carried out with an initial denaturation at 95 °C, followed by 40 cycles of denaturation at 95 °C, annealing/extension at an appropriate primer-specific temperature, and final melt curve analysis to verify product specificity. Relative gene expression was calculated using the comparative Ct (2^−ΔΔCt^) method and expressed as fold change relative to control cells. For each sample type, three or four biological replicates collected on independent days were analyzed, with at least two technical replicates for each biological replicate [33,34].

### 2.7. Cell Permeability Assay

EOMA cells were seeded on 0.4 μm polycarbonate Transwell filters in a 24-well filtration microplate and grown to confluence. Cells were treated with TMAO alone or in combination with the MCD, DPI, or WEHD overnight. Following treatment, FITC-dextran (70 kDa, 2.5 μmol/L) was added to the upper chamber and incubated for 2.5 h. The medium from the lower chamber was then collected, and fluorescence intensity was measured using a spectrofluorometer (BioTek Instruments Inc., Winooski, VT, USA) at an excitation wavelength of 494 nm and an emission wavelength of 521 nm [28]. Relative fluorescence intensity was used as an index of endothelial permeability under different conditions.

### 2.8. RNA Interference of gp91^phox^ and Caspase-1

Small interfering RNAs (siRNAs) for gp91^phox^ and caspase-1 were commercially obtained from Santa Cruz Biotechnology, Dallas, TX, USA. The sequences for gp91^phox^ and caspase-1 were confirmed to be effective in silencing the gp91^phox^ and caspase-1 genes by the company. siRNA (40 nM) was transfected with the silentfect Lipid Reagent (Bio-Rad, Hercules, CA, USA) as per the manufacturer’s instructions. RNA interference on the expression of the targeted proteins was examined by RT-qPCR analysis and biochemical assay and was quantified relative to control group.

### 2.9. Statistical Analysis

Data are presented as the mean ± standard error of the mean (SEM), where *n* represents the number of independent experiments. Statistical significance was determined using one-way analysis of variance (ANOVA) followed by Dunnett’s post hoc multiple-comparison test. A *p* value < 0.05 was considered statistically significant.

## 3. Results

### 3.1. TMAO Induces Membrane Raft Clustering in Endothelial Cells

**Figure 1:**
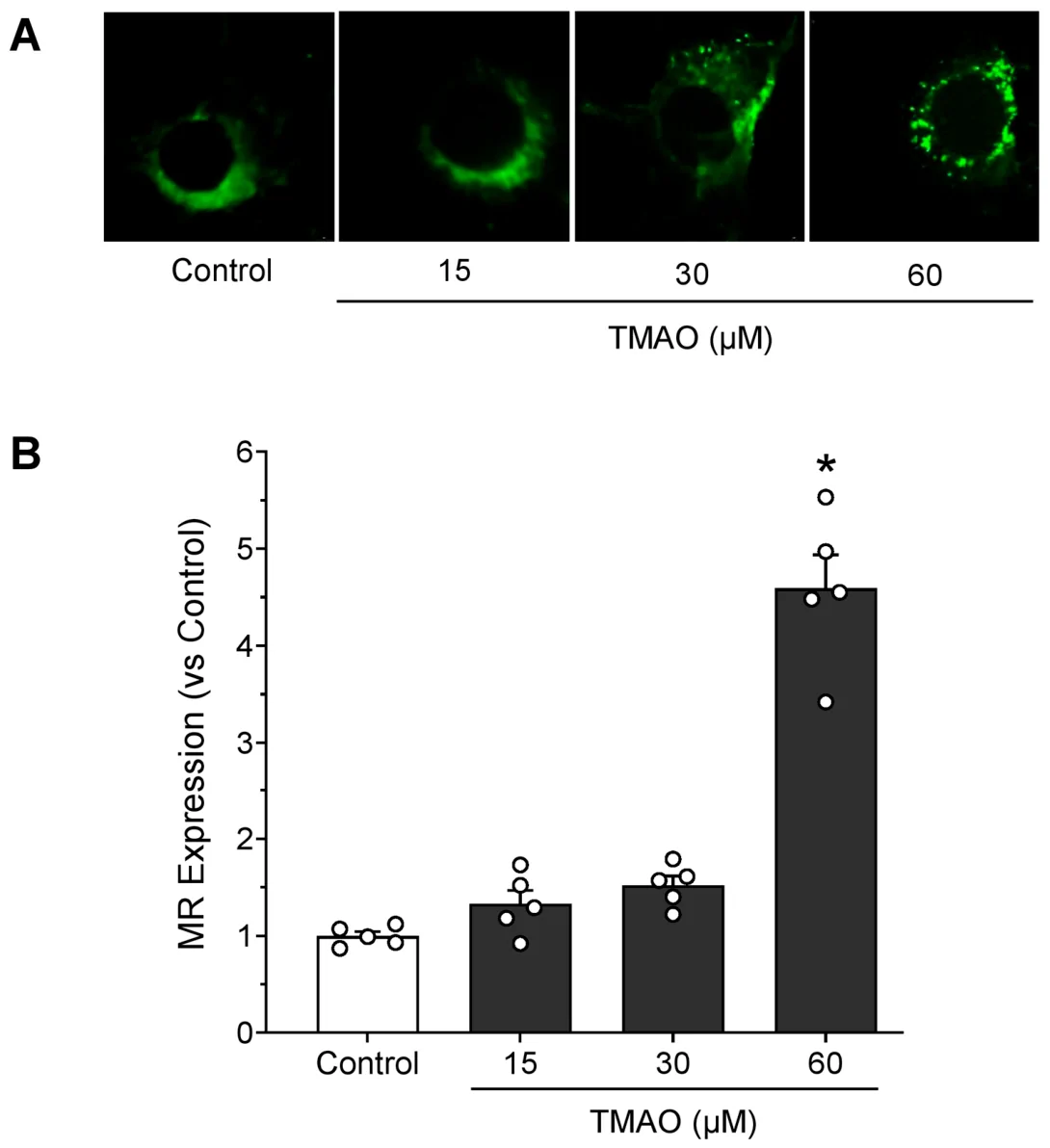
TMAO induces membrane raft clustering in endothelial cells. (**A**) Representative confocal images showing MR clustering in EOMA cells treated with TMAO at 15, 30, and 60 μM for 16 h. (**B**) Quantitative analysis of MR clustering relative to control cells. TMAO increased MR clustering in a dose-dependent manner, with the most marked effect observed at 60 μM. Data are presented as mean ± SEM. * *p* < 0.05 vs control.

To evaluate the effect of TMAO on MR clustering in endothelial cells, cultured EOMA cells were treated with TMAO at concentrations of 15, 30 and 60 μM for 16 h. Confocal microscopy analysis revealed a marked increase in MR clustering in cells treated with 60 μM TMAO compared with untreated controls (Figure 1A,B). Therefore, 60 μM TMAO was considered in all subsequent experiments.

### 3.2. TMAO Promotes the Colocalization of gp91phox and p47phox with Membrane Raft Clusters in Endothelial Cells

**Figure 2:**
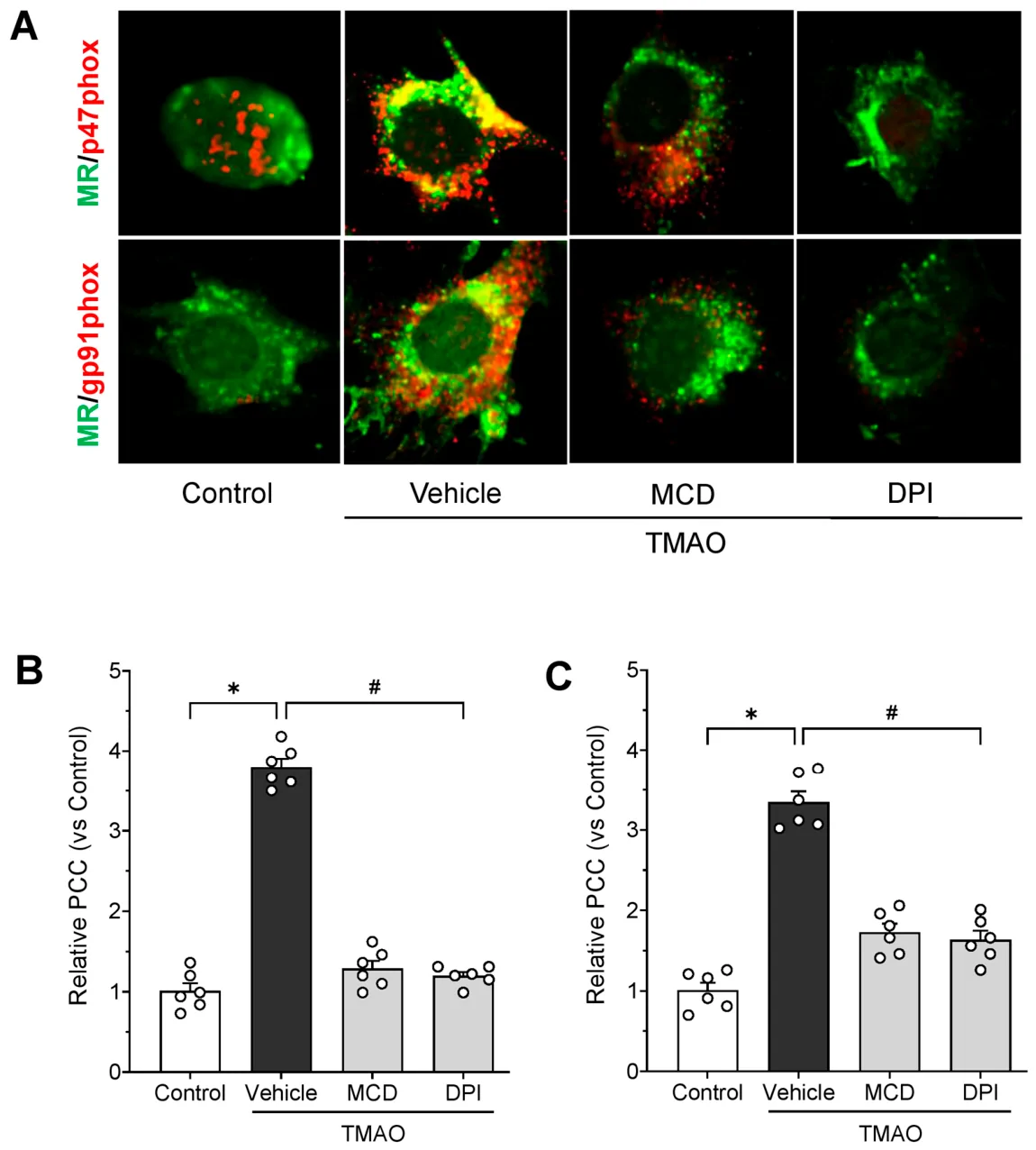
TMAO promotes the colocalization of membrane rafts with NADPH oxidase subunits in endothelial cells. (**A**) Representative confocal images showing colocalization of MRs (green) with p47phox or gp91phox (red) in control EOMA cells and in cells treated with TMAO (60 μM) alone or in combination with MCD or DPI. (**B**) Quantification of MR/p47phox colocalization by PCC. (**C**) Quantification of MR/gp91phox colocalization by PCC. TMAO increased colocalization of MRs with both NADPH oxidase subunits, whereas MCD and DPI attenuated these responses. Data are presented as mean ± SEM. * *p* < 0.05 vs. control; # *p* < 0.05 vs. TMAO.

To examine the effect of TMAO on MR clustering and its impact on NADPH oxidase subunits, EOMA cells were treated with TMAO, and the colocalization of gp91phox and p47phox with MRs was assessed by confocal microscopy using subunit-specific antibodies and Alexa Fluor 488-labeled CTxB. In untreated cells, CTxB staining showed a relatively uniform membrane distribution (Figure 2A). In contrast, TMAO treatment induced prominent MR clustering, which appeared as discrete fluorescent patches and showed increased colocalization with gp91phox and p47phox. To further evaluate the role of MRs and NADPH oxidase in this response, cells were pretreated with the MR disruptor MCD or the NADPH oxidase inhibitor DPI before TMAO stimulation. Both MCD and DPI markedly reduced the TMAO-induced colocalization of gp91phox and p47phox with MRs (Figure 2B,C). These findings suggest that TMAO promotes the formation of MR-associated redox signaling platforms in endothelial cells, accompanied by the recruitment of gp91phox and p47phox, and that this response is attenuated by disruption of MRs or inhibition of NADPH oxidase.

### 3.3. TMAO Induces Superoxide Production via NADPH Oxidase Activation

**Figure 3:**
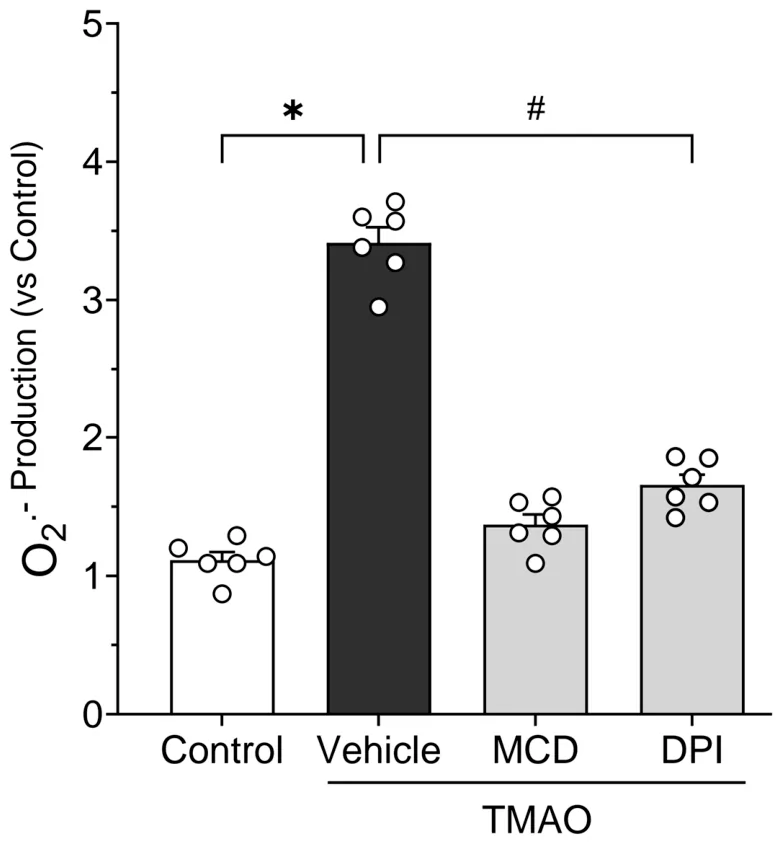
TMAO increases superoxide production through membrane raft-associated NADPH oxidase activation. Superoxide (O_2_·^−^) production was measured in EOMA cells by ESR. TMAO (60 μM) significantly increased O_2_·^−^ generation compared to the vehicle-treated control cells. Pretreatment with MCD or DPI attenuated the TMAO-induced increase in O_2_·^−^ production. Data are presented as mean ± SEM. * *p* < 0.05 vs. control; # *p* < 0.05 vs. TMAO.

To evaluate the role of NADPH oxidase in TMAO-induced superoxide (O_2_·^−^) production, we utilized ESR spectroscopy. TMAO treatment significantly elevated O_2_·^−^ levels in endothelial cells compared to vehicle-treated control cells (Figure 3). Pre-treatment with the MR disruptor MCD or the NADPH oxidase inhibitor DPI markedly reduced TMAO-induced O_2_·^−^ production, bringing the levels closer to those observed in control cells. These results indicate that TMAO enhances superoxide generation through an MR-associated, NADPH oxidase-dependent mechanism in endothelial cells.

### 3.4. TMAO-Induced NADPH Oxidase Activation Facilitates NLRP3 Inflammasome Formation in Endothelial Cells

**Figure 4:**
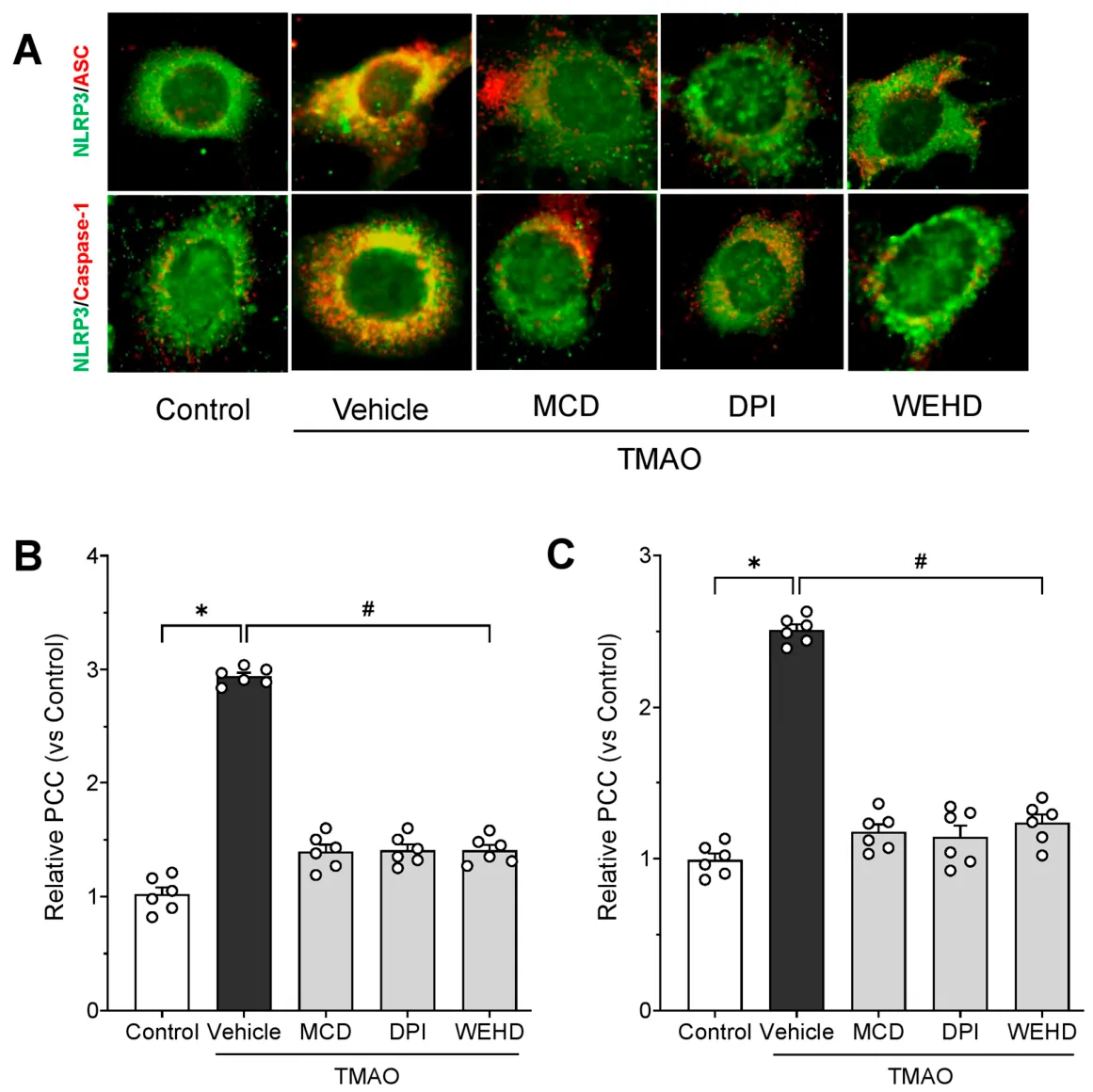
TMAO enhances NLRP3 inflammasome formation in endothelial cells. (**A**) Representative confocal images showing colocalization of NLRP3 (green) with ASC (red, upper panels) or caspase-1 (red, lower panels) in control EOMA cells and in cells treated with TMAO (60 μM) alone or in combination with MCD, DPI, or WEHD. Yellow fluorescence in merged images indicates colocalization. (**B**) Quantification of NLRP3/ASC colocalization by PCC. (**C**) Quantification of NLRP3/caspase-1 colocalization by PCC. TMAO increased inflammasome formation, whereas MCD, DPI, and WEHD attenuated these responses. Data are presented as mean ± SEM. * *p* < 0.05 vs. control; # *p* < 0.05 vs. TMAO.

To investigate whether TMAO-induced NADPH oxidase activation contributes to endothelial cell injury by promoting NLRP3 inflammasome formation, we analyzed the colocalization of NLRP3 inflammasome components in TMAO-treated endothelial cells using confocal microscopy. TMAO treatment significantly increased the colocalization of NLRP3 with ASC and NLRP3 with caspase-1, as evidenced by the increased yellow fluorescence in merged images (Figure 4A). Pretreatment with the membrane raft disruptor MCD, the NADPH oxidase inhibitor DPI, or the caspase-1 inhibitor WEHD attenuated the TMAO-induced colocalization of NLRP3 with ASC and NLRP3 with caspase-1 (Figure 4A–C). These results suggest that TMAO promotes NLRP3 inflammasome formation in endothelial cells and that this response is reduced by disruption of MRs, inhibition of NADPH oxidase, or blockade of caspase-1.

### 3.5. Targeting NADPH Oxidase and Membrane Rafts Attenuates TMAO-Induced Caspase-1 Activation and IL-1β Production in Endothelial Cells

**Figure 5:**
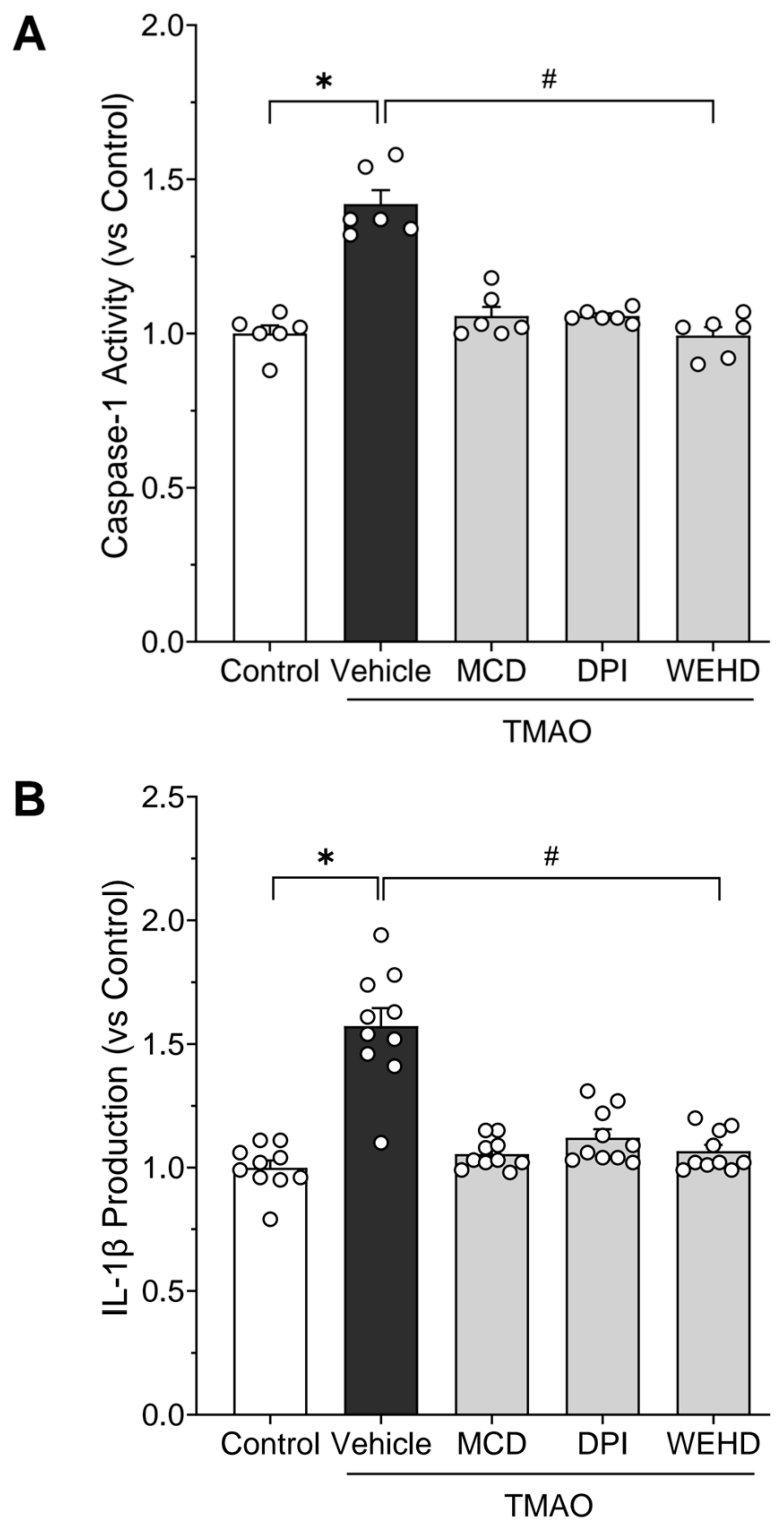
Targeting membrane rafts, NADPH oxidase, or caspase-1 attenuates TMAO-induced inflammasome-associated responses in endothelial cells. (**A**) Caspase-1 activity in EOMA cells treated with TMAO (60 μM) alone or in combination with MCD, DPI, or WEHD. (**B**) IL-1β production measured by ELISA under the indicated treatment conditions. TMAO increased caspase-1 activity and IL-1β production, whereas MCD, DPI, and WEHD significantly attenuated these effects. Data are presented as mean ± SEM. * *p* < 0.05 vs. control; # *p* < 0.05 vs. TMAO.

TMAO treatment significantly increased caspase-1 activity and IL-1β production in EOMA cells compared to vehicle-treated control cells (Figure 5A,B). Pretreatment with the membrane raft disruptor MCD, the NADPH oxidase inhibitor DPI, or the caspase-1 inhibitor WEHD significantly attenuated the TMAO-induced increase in caspase-1 activity and IL-1β production. These findings indicate that TMAO enhances inflammasome-associated responses in endothelial cells and that these effects are reduced by disruption of MRs, inhibition of NADPH oxidase, or blockade of caspase-1 activity. To further strengthen these pharmacological findings, we performed siRNA-mediated knockdown experiments targeting gp91phox (NOX2) and caspase-1. TMAO significantly increased caspase-1 activity compared to vehicle-treated control cells, whereas knockdown of either gp91phox or caspase-1 significantly attenuated the TMAO-induced increase in caspase-1 activity. These findings provide complementary genetic evidence that NOX2-dependent redox signaling contributes to downstream caspase-1 activation in response to TMAO (Supplemental Figure S1).

### 3.6. TMAO-Induced Endothelial Damage Reduces Junctional Protein Expression and Disrupts Endothelial Barrier Integrity

**Figure 6:**
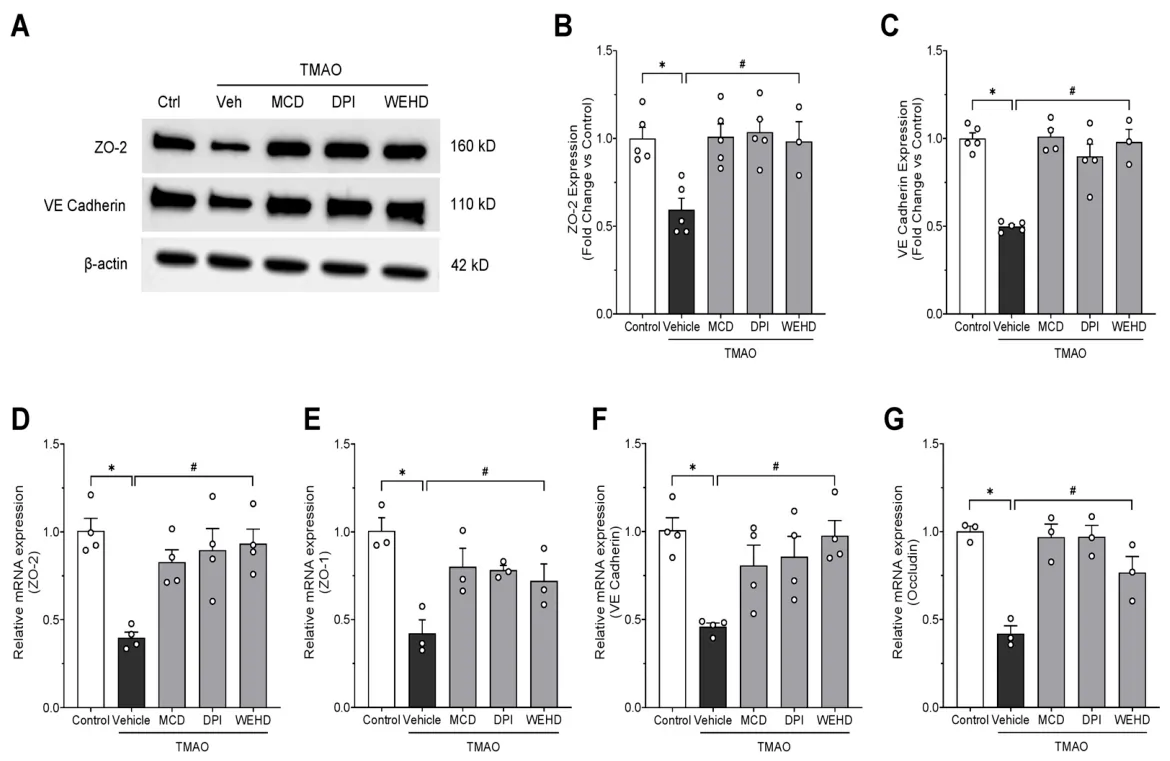
TMAO reduces endothelial junctional protein and gene expression in EOMA cells. (**A**) Representative Western blot analysis showing ZO-2 and VE-cadherin protein expression in control EOMA cells and in cells treated with TMAO (60 μM) alone or in combination with MCD, DPI, or WEHD. β-actin was used as the loading control (*n* = 3–5). (**B**,**C**) Quantification of ZO-2 and VE-cadherin protein expression normalized to β-actin and expressed relative to control (n = 3–5). (**D**–**G**) Relative mRNA expression of ZO-2, ZO-1, VE cadherin and occludin determined by RT-qPCR. TMAO significantly reduced ZO-2, ZO-1, VE cadherin and occludin expressions, whereas MCD, DPI, and WEHD attenuated these effects. Data are presented as mean ± SEM. * *p* < 0.05 vs. control; # *p* < 0.05 vs. TMAO.

To assess endothelial junction injury, we examined the expression of junctional protein markers ZO-2, ZO-1, VE cadherin and occludin in EOMA cells. ZO-2, ZO-1, VE cadherin and occludin play important roles in maintaining endothelial junction organization and barrier integrity. Western blot analysis showed that TMAO treatment markedly reduced ZO-2 and VE cadherin protein expression compared to control cells (Figure 6A). Quantitative analysis further confirmed a significant decrease in ZO-2 and VE cadherin protein expression following TMAO treatment (Figure 6B,C). Pretreatment with the MR disruptor MCD, the NADPH oxidase inhibitor DPI, or the caspase-1 inhibitor WEHD significantly attenuated the TMAO-induced reduction in ZO-2 and VE cadherin protein expression (Figure 6B,C). Further, RT-qPCR analysis also demonstrated that TMAO significantly reduced ZO-2, ZO-1, VE cadherin, and occludin mRNA expression (Figure 6D–G). Pretreatment with the MR disruptor MCD, the NADPH oxidase inhibitor DPI, or the caspase-1 inhibitor WEHD significantly attenuated these TMAO-induced reductions and preserved ZO-2, ZO-1, VE cadherin and occludin expression in TMAO-treated cells. These findings indicate that TMAO disrupts endothelial junctional protein expression and that this effect is attenuated by membrane raft disruption, NADPH oxidase inhibition, or caspase-1 inhibition.

To further support the involvement of NOX2/gp91phox and caspase-1 in TMAO-induced endothelial injury, we examined the effects of siRNA-mediated knockdown of gp91phox and caspase-1. TMAO significantly reduced VE-cadherin and ZO-1 mRNA expressions compared with control cells. Importantly, knockdown of either gp91phox or caspase-1 significantly attenuated these TMAO-induced reductions and preserved VE-cadherin and ZO-1 expression (Supplementary Figure S2). These findings provide complementary genetic evidence that NOX2/gp91phox-dependent redox signaling and downstream caspase-1 activation contributes to TMAO-induced disruption of endothelial junction protein expression.

### 3.7. TMAO-Induced NLRP3 Inflammasome Activation Disrupts Endothelial Barrier Function

**Figure 7:**
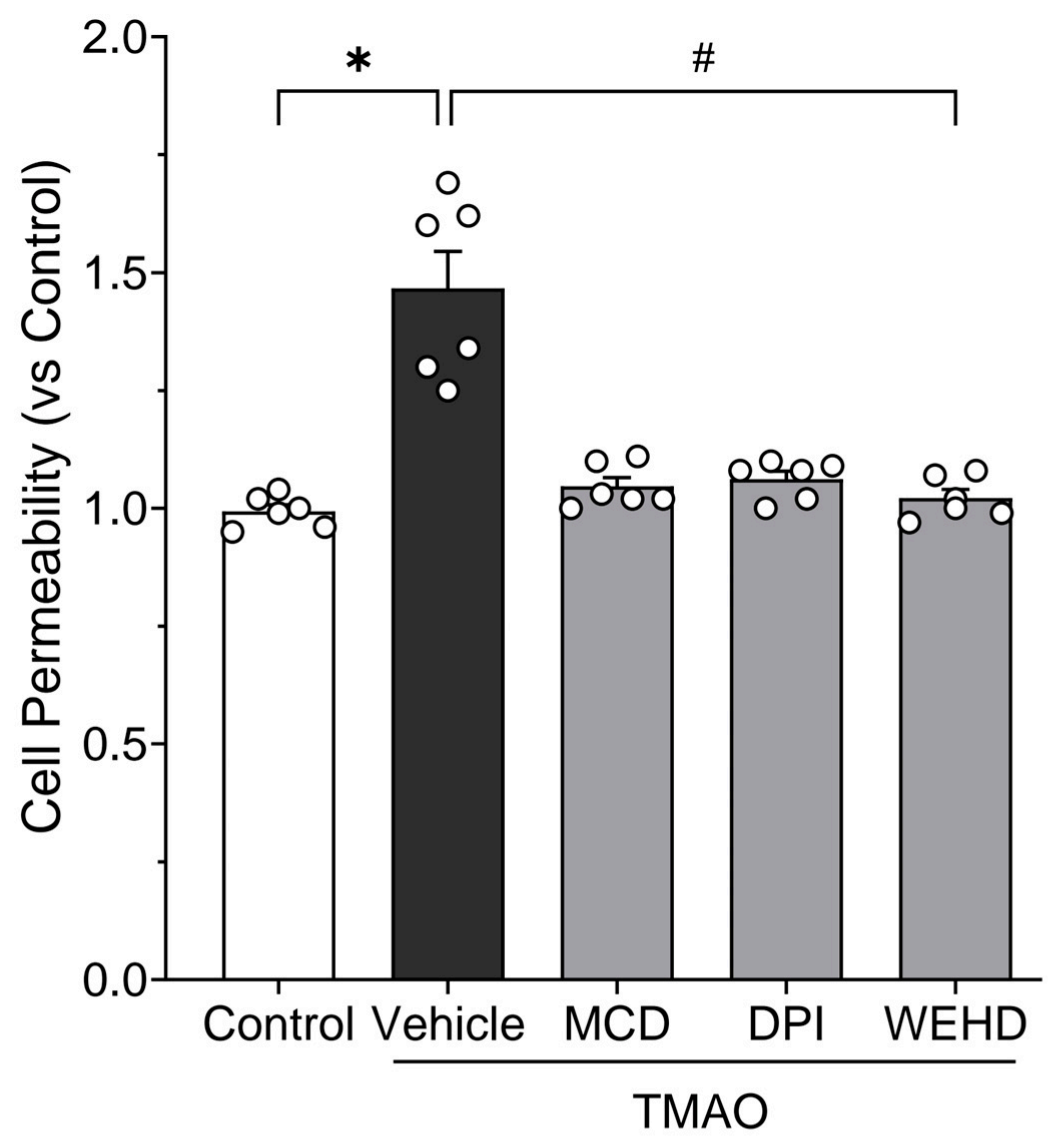
TMAO disrupts endothelial barrier function. Endothelial permeability was assessed in EOMA monolayers using FITC-dextran. TMAO significantly increased endothelial permeability compared with vehicle-treated control cells. Pretreatment with MCD, DPI, or WEHD attenuated the TMAO-induced increase in permeability. Data are presented as mean ± SEM. * *p* < 0.05 vs. control; # *p* < 0.05 vs. TMAO.

TMAO treatment significantly increased endothelial cell permeability compared with vehicle-treated control cells (Figure 7), indicating impaired barrier function. Pretreatment with the MR disruptor MCD, the NADPH oxidase inhibitor DPI, or the caspase-1 inhibitor WEHD significantly attenuated the TMAO-induced increase in permeability. These findings indicate that TMAO impairs endothelial barrier function and that this response is reduced by MR disruption, inhibition of NADPH oxidase, or blockade of caspase-1.

## 4. Discussion

The present study provides evidence that membrane raft (MR)-associated redox signaling is a key mechanism in TMAO-induced endothelial dysfunction. TMAO increased MR clustering, promoted the recruitment of NADPH oxidase subunits to raft domains, enhanced superoxide generation, and stimulated NLRP3 inflammasome formation in endothelial cells. These changes were accompanied by increased caspase-1 activity, elevated IL-1β production, reduced junction protein expression, and increased endothelial permeability. Collectively, these findings support a model in which MR-dependent redox signaling contributes to the pro-inflammatory and barrier-disrupting effects of TMAO.

The NLRP3 inflammasome is a multiprotein complex that plays a pivotal role in the innate immune response by mediating the production of pro-inflammatory cytokines such as IL-1β and IL-18 [23,35]. Its activation is triggered by diverse stimuli, including ROS, lysosomal damage, and cellular stress, positioning it as a critical link between metabolic disturbances and inflammation [23,36]. In endothelial dysfunction, NLRP3 inflammasome activation drives vascular inflammation, increased endothelial permeability, and the disruption of barrier integrity, all of which are hallmarks of atherosclerosis and cardiovascular diseases [4,37,38].

TMAO, a metabolite derived from dietary nutrients by gut microbiota, has been implicated in the pathogenesis of cardiovascular diseases (CVDs) through mechanisms such as oxidative stress, inflammation, and endothelial barrier disruption [39]. Elevated TMAO levels are strongly associated with a heightened risk of major adverse cardiovascular events, including myocardial infarction and stroke [8,40,41]. Our previous work showed that TMAO activates the NLRP3 inflammasome and impairs endothelial function [25]. The present study is the first to demonstrate the upstream signaling mechanisms linking TMAO exposure to inflammasome activation and subsequent barrier dysfunction.

Correspondingly, MRs are cholesterol-rich microdomains that function as signaling platforms in endothelial cells [16,17]. In the present study, TMAO enhanced MR clustering and increased the colocalization of gp91phox and p47phox with raft domains. These findings suggest that TMAO promotes the assembly of an MR-associated redox signaling platform. Disruption of MRs with MCD markedly reduced this response. A similar effect was observed with DPI. Together, these results indicate that MR integrity is required for efficient recruitment of NADPH oxidase components under TMAO stimulation. In particular, the NADPH oxidase is a major source of reactive oxygen species in endothelial cells and has a central role in vascular injury [20,22,23]. In our study, TMAO markedly increased superoxide production, and this increase was attenuated by MCD and DPI. These data indicate that TMAO-induced oxidative stress is closely linked to MR-dependent NADPH oxidase activation. Because ROS are known to promote inflammasome activation and impair junctional stability, enhanced redox signaling is likely an important event in the development of TMAO-induced endothelial dysfunction.

The present findings further support a role for the NLRP3 inflammasome in this process. TMAO increased the colocalization of NLRP3 with ASC and caspase-1, indicating enhanced inflammasome assembly. TMAO also increased caspase-1 activity and IL-1β production, which is consistent with inflammasome activation in endothelial cells. Importantly, these responses were reduced by MCD, DPI and WEHD, supporting the interpretation that MR-dependent redox signaling acts upstream of inflammasome activation. The outcomes also define an important downstream role for caspase-1. WEHD, a caspase-1 inhibitor, reduced NLRP3-ASC and NLRP3-caspase-1 colocalization, decreased caspase-1 activity and IL-1β production, and attenuated the loss of junctional proteins and the increase in endothelial permeability. These findings indicate that caspase-1 activation is not merely a marker of inflammasome assembly, but a functional mediator of endothelial injury in response to TMAO. The protective effect of WEHD places caspase-1 downstream of MR-associated redox signaling and upstream of barrier disruption.

Notably, earlier studies identified TMAO as a key contributor to the disruption of endothelial monolayer integrity, resulting in increased permeability [25]. The endothelium functions as a crucial barrier between the vascular lumen and surrounding tissues, playing an essential role in regulating fluid homeostasis, angiogenesis, and vascular tone [42]. This barrier’s integrity relies on tight and adherent junction proteins, such as ZO-2, ZO-1, VE cadherin and occludin, which regulate inter-endothelial junction permeability under physiological and pathological conditions [43,44,45,46,47]. The disruption of these proteins is a defining feature of endothelial dysfunction and is implicated in numerous pathological states, including chronic kidney disease, venous thrombosis, stroke, cardiovascular disease, diabetes, tumor metastasis, and certain viral infections [43,48,49]. In the present study, TMAO reduced the expression of tight and adherent junction proteins and increased endothelial permeability. These observations are consistent with earlier reports that TMAO compromises endothelial barrier integrity [25]. Importantly, MCD, DPI, and WEHD attenuated these effects. This pattern supports the view that TMAO disrupts endothelial junctions through a pathway involving MR clustering, NADPH oxidase-derived oxidative stress, and caspase-1-dependent inflammasome signaling. Correspondingly, the RT-qPCR results further strengthened this interpretation at the transcriptional level. TMAO significantly reduced the mRNA expression of ZO-2 and ZO-1, and these decreases were attenuated by MCD, DPI, or WEHD. These findings suggest that the loss of endothelial barrier integrity after TMAO exposure is associated not only with reduced junction protein abundance, but also with downregulation of genes required for tight and adherent junction maintenance. In addition, the observed changes in VE cadherin and occludin mRNA expression are consistent with the same overall pattern. Together, these data indicate that TMAO impairs endothelial integrity at both the protein and transcriptional levels through an MR-NADPH oxidase-caspase-1-dependent mechanism.

A relevant consideration is the TMAO concentration used in the present study. The 60 µM stimulus employed here is higher than the basal fasting plasma concentrations commonly reported in healthy individuals and in many cardiovascular cohorts. For example, in patients undergoing elective coronary angiography, the highest TMAO quartile was defined as >6.18 µM and was associated with an increased risk of major adverse cardiovascular events [50]. However, circulating TMAO concentrations increase substantially in renal dysfunction. In patients with chronic kidney disease, median plasma TMAO levels of 7.9 µM (IQR, 5.2–12.4 µM) have been reported [51], whereas dialysis-dependent patients showed median concentrations of 94.4 µM (IQR, 54.8–133.0 µM), a range that encompasses the 60 µM concentration used in the present study [52]. Therefore, 60 µM TMAO should not be interpreted as representative of basal circulating exposure in healthy individuals, but rather as an in vitro concentration within the range documented under severe pathophysiological conditions, particularly advanced renal dysfunction. This concentration was selected based on the dose–response data in Figure 1, where it produced a consistent and reproducible increase in membrane raft clustering in EOMA cells, and it is consistent with concentrations used in prior work from our laboratory using the same cell model [28]. Whether this MR/NADPH oxidase/NLRP3/caspase-1 signaling axis is engaged at lower circulating TMAO concentrations or under in vivo pathophysiological conditions remains to be established. The present study has several limitations. First, all experiments were conducted using cultured endothelial cells; therefore, our conclusions are limited to an in vitro model. Further studies using appropriate animal models are warranted to determine whether the membrane raft-mediated redox signaling mechanisms identified here similarly contribute to TMAO-induced NLRP3 inflammasome activation and endothelial dysfunction in vivo. Second, although our pharmacological and genetic data support the involvement of membrane raft-associated NADPH oxidase signaling, pharmacological agents such as DPI and MCD have recognized limitations, including off-target effects and nonspecific disruption of membrane-associated signaling pathways. Importantly, our additional siRNA experiments targeting NOX2/gp91phox and caspase-1 provided complementary genetic evidence supporting the involvement of this pathway. Nevertheless, future studies employing more selective NOX inhibitors and additional genetic approaches, including knockdown or knockout of NOX2 (gp91phox), p47phox, or other relevant NADPH oxidase isoforms, will be important to further define the specific contributions of individual NADPH oxidase isoforms and membrane raft signaling to TMAO-induced NLRP3 inflammasome activation and endothelial dysfunction. Third, although our imaging and co-localization studies provide evidence for the spatial association of membrane raft domains with NADPH oxidase and downstream inflammatory signaling, membrane raft isolation and immunoprecipitation-based studies would provide more direct biochemical evidence for the composition and molecular organization of the proposed signaling complex. Future biochemical studies will therefore be important to directly define the molecular interactions and organization of the membrane raft-associated signaling complex in response to TMAO.

## 5. Conclusions

This investigation identifies MR-associated NADPH oxidase redox signaling and downstream NLRP3 inflammasome activation as central mechanisms underlying TMAO-induced endothelial dysfunction. TMAO promoted MR clustering, enhanced NADPH oxidase-dependent superoxide generation, stimulated inflammasome activation, and reduced junction protein expression at both the protein and mRNA levels and increased endothelial permeability. These results indicate that oxidative stress and inflammasome signaling are closely involved in TMAO-mediated vascular barrier injury. Moreover, the protective effects of MCD, DPI, and WEHD support the functional importance of MRs, NADPH oxidase activity, and caspase-1 activation in this process. Together, these findings provide a mechanistic framework for understanding how a gut microbiota-derived metabolite disrupts endothelial integrity and promotes inflammatory vascular injury. These outcomes also provide a basis for future studies aimed at targeting this signaling axis to limit endothelial dysfunction and related cardiovascular complications.

## Data Availability

The data presented in this study are available upon request from the corresponding author.

## Supplementary Materials

The following supporting information can be downloaded at: https://www.mdpi.com/article/10.3390/antiox15091138/s1, Figure S1: Targeting NADPH oxidase, or caspase-1 attenuates TMAO-induced caspase-1 activity in endothelial cells. Caspase-1 activity in EOMA cells treated with TMAO (60 μM) alone or in combination with gp91phox siRNA and caspase-1 siRNA. Data are presented as mean ± SEM. * *p* < 0.05 vs. control; # *p* < 0.05 vs. TMAO; Figure S2: TMAO decreases VE cadherin and ZO-1 expression in endothelial cells. Relative mRNA expression of VE cadherin (A) and ZO-1 (B) determined by RT-qPCR. TMAO significantly reduced VE cadherin and ZO-1 mRNA expression, whereas gp91phox siRNA and caspase-1 siRNA attenuated these effects. Data are presented as mean ± SEM. * *p* < 0.05 vs. control; # *p* < 0.05 vs. TMAO.

## Abbreviations

The following abbreviations are used in this manuscript:

ASC	apoptosis-associated speck-like protein containing a CARD
ANOVA	analysis of variance
BSA	bovine serum albumin
CMH	1-hydroxy-3-methoxycarbonyl-2,2,5,5-tetramethylpyrrolidine
CO_2_	carbon dioxide
CTxB	cholera toxin B
DMEM	Dulbecco’s Modified Eagle Medium
DPI	diphenyleneiodonium
ELISA	enzyme-linked immunosorbent assay
EOMA	mouse endothelial hemangioendothelioma cells
ESR	electron spin resonance
FBS	fetal bovine serum
FITC	fluorescein isothiocyanate
FMO3	flavin-containing monooxygenase 3
gp91phox	glycoprotein 91phox
IL-1β	interleukin-1 beta
MCD	methyl-β-cyclodextrin
MR	membrane raft
NADPH	nicotinamide adenine dinucleotide phosphate
NLRP3	NOD-like receptor family pyrin domain containing 3
O_2_·^−^	superoxide
PBS	phosphate-buffered saline
PCC	Pearson correlation coefficient
ROS	reactive oxygen species
RT-qPCR	quantitative reverse transcription polymerase chain reaction
SEM	standard error of the mean
SOD	superoxide dismutase
TMAO	trimethylamine-N-oxide
ZO-1	zonula occludens-1
ZO-2	zonula occludens-2
