# Supplementary material for: Membrane Raft Redox Signaling Mediates Trimethylamine N-Oxide-Induced NLRP3 Inflammasome Activation and Endothelial Dysfunction
Source: Antioxidants (Basel). 2026 Sep 9;15(9):1138. doi: 10.3390/antiox15091138 (PMC13603778; doi:10.3390/antiox15091138)
Supplement: Supplementary file 1 [file antioxidants-15-01138-s001.zip › antioxidants-4389893-supplementary.pdf]

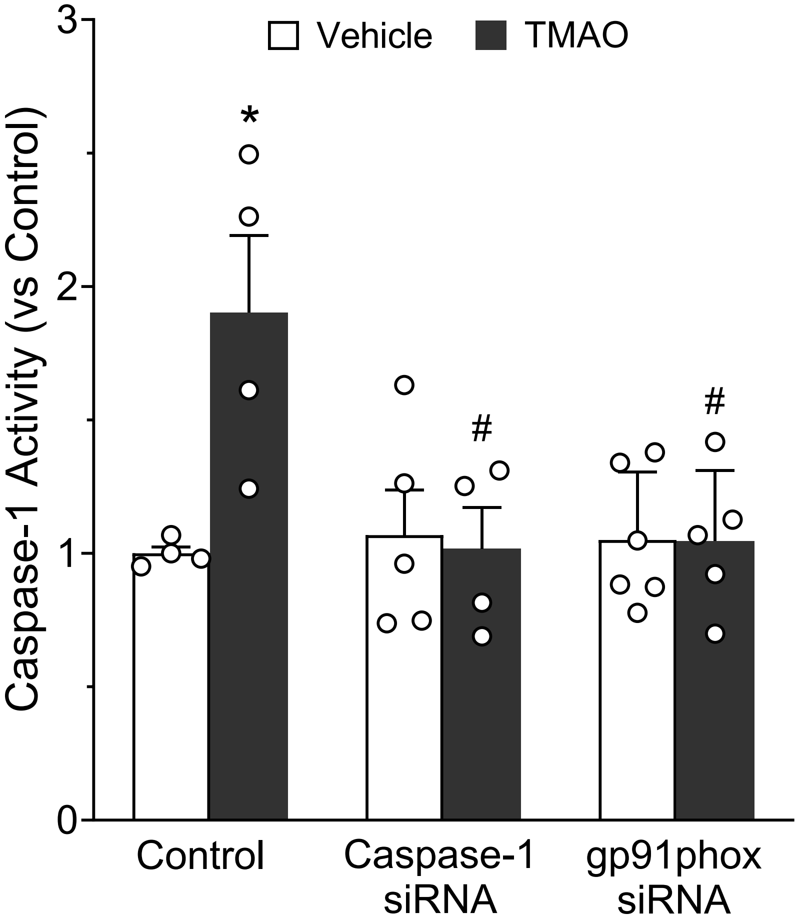

Supplemental Figure S1. Targeting NADPH oxidase, or caspase-1 attenuates TMAO-induced caspase-1 activity in endothelial cells. Caspase-1 activity in EOMA cells treated with TMAO (60 μM) alone or in combination with gp91phox siRNA and caspase-1 siRNA. Data are presented as mean ± SEM. **P < 0.05* vs control; *#P < 0.05* vs TMAO.

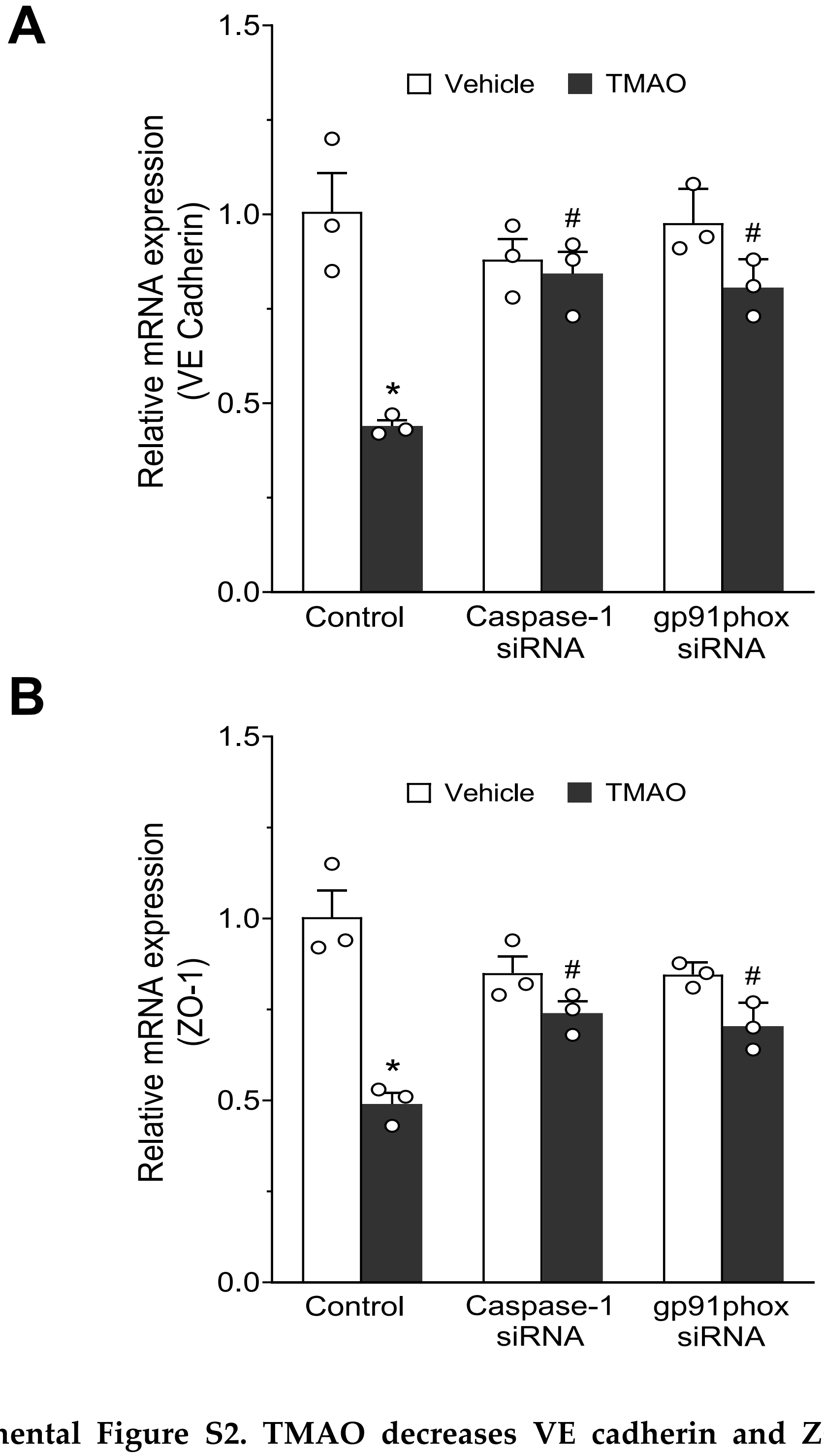

Supplemental Figure S2. TMAO decreases VE cadherin and ZO-1 expression in endothelial cells. Relative mRNA expression of VE cadherin (A) and ZO-1 (B) determined by RT-qPCR. TMAO significantly reduced VE cadherin and ZO-1 mRNA expression, whereas gp91phox siRNA and caspase-1 siRNA attenuated these effects. Data are presented as mean ± SEM. *$P < 0.05$ vs control; #$P < 0.05$ vs TMAO.
